# Infectious Diseases Associated with Hydrometeorological Hazards in Europe: Disaster Risk Reduction in the Context of the Climate Crisis and the Ongoing COVID-19 Pandemic

**DOI:** 10.3390/ijerph191610206

**Published:** 2022-08-17

**Authors:** Maria Mavrouli, Spyridon Mavroulis, Efthymios Lekkas, Athanassios Tsakris

**Affiliations:** 1 Department of Microbiology, Medical School, National and Kapodistrian University of Athens, 11527 Athens, Greece; 2 Department of Dynamic Tectonic Applied Geology, Faculty of Geology and Geoenvironment, School of Sciences, National and Kapodistrian University of Athens, 15784 Athens, Greece

**Keywords:** infectious disease, floods, waterborne disease, rodent-borne disease, vector-borne disease, outbreak, multi-hazard, disaster management, disaster risk reduction

## Abstract

Hydrometeorological hazards comprise a wide range of events, mainly floods, storms, droughts, and temperature extremes. Floods account for the majority of the related disasters in both developed and developing countries. Flooding alters the natural balance of the environment and frequently establish a favorable habitat for pathogens and vectors to thrive. Diseases caused by pathogens that require vehicle transmission from host to host (waterborne) or a host/vector as part of their life cycle (vector-borne) are those most likely to be affected by flooding. Considering the most notable recent destructive floods events of July 2021 that affected several Central Europe countries, we conducted a systematic literature review in order to identify documented sporadic cases and outbreaks of infectious diseases in humans in Europe, where hydrometeorological hazards, mainly floods, were thought to have been involved. The occurrence of water-, rodent-, and vector-borne diseases in several European countries is highlighted, as flooding and the harsh post-flood conditions favor their emergence and transmission. In this context, strategies for prevention and management of infectious disease outbreaks in flood-prone and flood-affected areas are also proposed and comprise pre- and post-flood prevention measures, pre- and post-outbreak prevention measures, as well as mitigation actions when an infectious disease outbreak finally occurs. Emphasis is also placed on the collision of floods, flood-related infectious disease outbreaks, and the evolving COVID-19 pandemic, which may result in unprecedented multi-hazard conditions and requires a multi-hazard approach for the effective disaster management and risk reduction.

## 1. Introduction

Hydrometeorological hazards comprise a wide range of events, mainly floods, storms, droughts, and temperature extremes along with their cascading effects. Despite their atmospheric, hydrological, or oceanographic origin, they can have considerable impact to hazards belonging to other categories, such as biological health hazards (e.g., infectious disease outbreaks and epidemics). Hydrometeorological hazards are characterized by high potential to adversely affect the structural environment including buildings and infrastructures, but also the public health in several ways.

Based on the International Disaster Database EM-DAT compiled by the Center for research on the Epidemiology of Disasters (CRED), one of the foremost international databases of such events, floods, and storms holds the highest number of occurrences during the last 30 years (1990–2020) [1]. As regards 2020, floods accounted for 51.67% of all incidents (ranking first), 40.92% of all fatalities (ranking second after extreme temperatures), 33.71% of the population affected (ranking second after storms), and 29.95% of economic losses in billion USD (ranking second after storms) by all disasters caused by natural hazards worldwide [2]. Taking into consideration the aforementioned numbers and percentages, it is obvious that floods account for the majority of the related disasters in both developed and developing countries.

Various definitions of floods have been provided from several sources [3,4,5,6]. However, their common point is the temporary partial or complete water inundation of normally dry land resulting from various processes and leading to loss of human life, dramatic effects on the natural environment, and considerable damage to the built environment. Regardless of classifications, floods have a wide range of health consequences that can be characterized in terms of time [7]. The immediate health effects of floods include drowning, injury, gastroenteritis outbreaks, and skin and respiratory infections, while the medium-term ones comprise of infected wounds, injury complications, poisoning, starvation and communicable diseases [8]. In the long-term, chronic disease, disability, poor mental health, and poverty-related diseases including malnutrition are usually recorded [8].

Infectious disease spread in populations is a consequence of the interaction and connection between the three components of the Epidemiologic Triangle: an external agent, a susceptible host, and an environment in which the agent and the host are brought together [9]. A vector that transfers the pathogen from one host to another without causing the disease itself could be involved in the infectious disease [10]. However, the effects of each triad component may vary substantially for different settings. Flooding alters the natural balance of the environment and frequently establishes a favorable habitat (breeding ground) for pathogens and vectors to thrive. Diseases caused by pathogens that require vehicle transmission from host to host (waterborne) or a host/vector as part of their life cycle (vector-borne) are those most likely to be affected by flooding [7,11,12].

**Figure 1:**
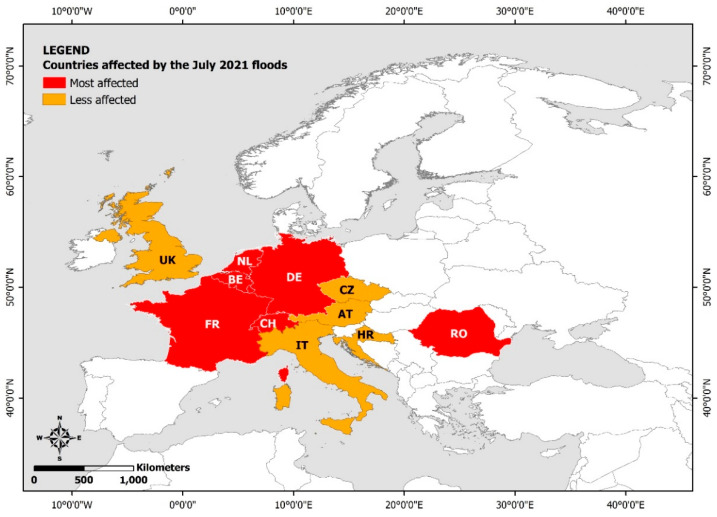
European countries affected by the July 2021 floods.

Since early 2020, amid the evolving COVID-19 pandemic and the ongoing climate crisis, which increase the frequency and magnitude of extreme weather events, floods of various magnitudes have been generated in many countries worldwide (e.g., [1,13,14,15,16,17]) including Europe. Among the most notable recent destructive events are the July 2021 floods that occurred in and affected several Central Europe countries (western Germany and the neighboring Belgium, Luxemburg, the Netherlands, and Switzerland were the most affected; Figure 1). Excessive rainfall along with saturated soils caused rivers to overflow their banks, resulting in extreme floods and devastation. Several urban and rural residential areas were inundated. Flooding led to significant damage to buildings and business property or equipment as well as destruction of crops and farms. Large parts of the road and railway network were submerged and bridges collapsed resulting in traffic and communication disruption. The water supply network was also compromised, thus negatively affecting the safety of drinking water and causing supply interruption and water shortage in the affected residential areas. The electric power supply network suffered damage to its structural elements, leaving hundreds of thousands households without electricity, telephone, and internet, and making the 112 Emergency Communications Service inaccessible. During intense flooding, the capacity of sewerage was exceeded, thus the sewer systems overflowed and inundated streets and buildings with raw sewage. More than 243 fatalities were reported (196 in Germany, 43 in Belgium, 2 in Romania, 1 in Italy, and 1 in Austria) and attributed to the aforementioned adverse effects of inundation and flooding. About 1000 residents were stranded in inaccessible areas for days, while hundreds of thousands had to evacuate flood-affected areas.

Apart from the immediate impact of the July 2021 Europe floods on human lives and infrastructure, these events also revealed another major public health concern: the risk of infectious disease outbreaks during and after floods. For example, flood-induced population displacement to emergency shelters may occur during and after a flood, depending on its magnitude and intensity as well as on the extent of the affected area. Conditions including overcrowding, poor sanitation, and lack of access to drinking water are all risk factors to which displaced populations are regularly exposed. This, combined with the disruption of health and sanitation services, raises the risk of displaced populations being exposed to several health and safety risks.

This natural hazard with its widespread impact on several sectors of daily life and in particular the high risk to public health in developed European countries prompted us to conduct an extensive and systematic review of the existing literature in order to present in detail the infectious diseases associated with hydrometeorological hazards in Europe that may occur in similar future events in heavily affected areas, resulting in extensive and prolonged disaster response and recovery. Amid the evolving COVID-19 pandemic and the ongoing climate crisis, the extensive knowledge and the understanding of the health risks are very crucial for public health, as the synergy of these outbreaks could create complex emergencies involving not only unprecedented conditions but also challenges in disaster management and disaster risk reduction.

## 2. Materials and Methods

In March 2022, all major databases and sources for medical, scientific, and technical research contained in the National Center for Biotechnology Information, part of the United States National Library of Medicine (NLM), were thoroughly searched to identify documented sporadic cases and outbreaks of infectious diseases in humans in Europe since 1910, where hydrometeorological hazards, mainly floods along with their accompanied phenomena, were thought to have been involved. More specifically, key word searches were carried out in PubMed, PubMed Health, PubMed Central, Scopus, and ScienceDirect.

Search terms were based on a Centers for Disease Control and Prevention (CDC) list of general resources related to possible health concerns associated with floods and the World Health Organization (WHO) fact sheet “Flooding and Communicable Diseases: risk assessment and preventive measures” [18]. A list of known pathogens was compiled and used to generate key search terms for the identification of infectious diseases related to flooding, usually as a result of heavy rainfall. All papers with the specified search terms in their titles, abstracts, or key words were searched for. For incorporating scientific journal articles and official reports not included in the aforementioned databases, an online search using related key phrases and their combinations was conducted using Google advanced search and Google Scholar.

Inclusion criteria were as follows: (a) Literature type: published articles and official reports in English; (b) natural disaster: flooding following mainly heavy rainfall; (c) population: human; (d) country: European countries; and (e) outcome measure: incidence increase or outbreak of infectious disease related to flooding. No standard definition of what constituted an outbreak was employed to avoid omitting potentially relevant studies of public health importance. In addition to being inclusive of a wide range of studies, there were no filters employed to identify specific study designs. Exclusion criteria were as follows: (a) literature type: news articles, (b) natural disaster: events other than floods, (c) population: non-human, (d) country: countries outside Europe, (e) outcome measure: incidence increase or outbreak of infectious diseases associated with natural hazards other than floods.

## 3. Results

### 3.1. Study Selection

**Figure 2:**
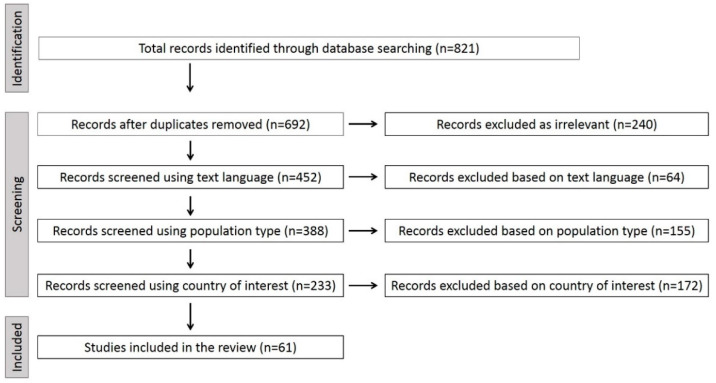
The study selection diagram showing the flow of documents through the literature review.

The initial search generated 821 records. Following the exclusion of duplicate entries and irrelevant articles, 452 records remained. Sixty four studies were not available in English. Thus, 388 articles were scanned based on population type and country of interest and were processed in more detail for eligibility. After the full text screening, a total of 61 articles were found to fit the inclusion criteria and were included in the analysis. The following study selection flow chart presents our results in detail (Figure 2).

Following the completion of the literature search and application of the inclusion criteria, the analyzed published articles focused on the most common infectious diseases and health risks associated with flood-affected areas in the United Kingdom, Denmark, France, Italy, Finland, Republic of Ireland, Germany, Greece, Sweden, the Czech Republic, the Netherlands, Spain, Hungary, Bulgaria, Norway, Romania, Bosnia and Herzegovina, Croatia, Montenegro, North Macedonia, and Serbia (Tables S1–S3).

In Europe, flooding, as well as the harsh post-flood conditions have been shown to favor the emergence and transmission of water-, rodent-, and vector-borne diseases, which are thoroughly described below. Of the 61 articles identified, 43 (70.5%) deal with the occurrence of waterborne diseases (Table S1), followed by 12 studies (19.7%) on the emergence of rodent-borne diseases (Table S2) and 6 (9.8%) studies on vector-borne diseases (Table S3).

### 3.2. Waterborne Diseases

**Figure 3:**
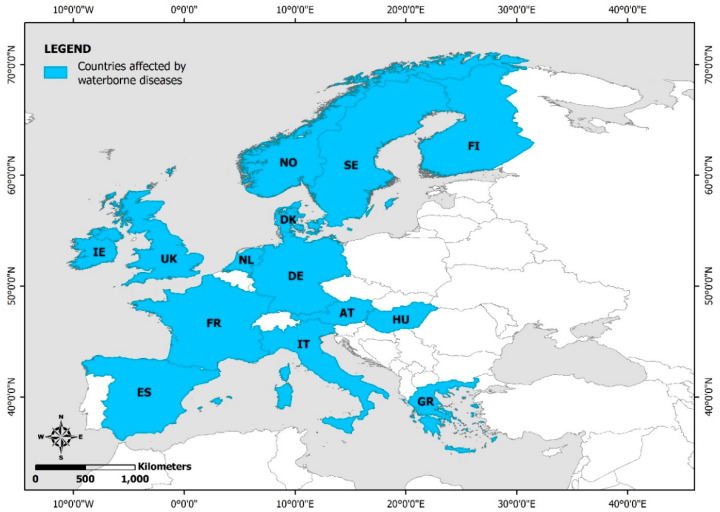
European countries with reported flood-related waterborne diseases (NO: Norway; SE: Sweden; FI: Finland; DK: Denmark; NL: Netherlands; IE: Republic of Ireland; UK: United Kingdom; ES: Spain; FR: France; DE: Germany; AT: Austria; HU: Hungary; IT: Italy; GR: Greece).

Waterborne diseases are mainly caused by drinking water contaminated with pathogenic microorganisms (bacteria, viruses and parasites) originating from human or animal feces. Flood-related waterborne diseases have been reported in the United Kingdom, the Republic of Ireland, Norway, Sweden, Finland, Denmark, the Netherlands, Austria, Hungary, France, Spain, Germany, Italy, and Greece (Figure 3).

A case-crossover study analyzed rainfall-induced drinking water-related outbreaks reported in England and Wales for the period extending from 1910 to 1999 following both low and excessive rainfall [19]. Various parasites and bacteria such as *Giardia*, *Cryptosporidium*, *Escherichia coli*, *Salmonella typhi*, *Salmonella paratyphi*, *Campylobacter*, and *Streptobacillus moniliformis* were among the pathogens implicated in 89 outbreaks studied [19]. Street flooding and/or flooding of combined sewerage systems contribute to the mixing of rainwater with sewage, significantly contaminating flood waters with fecal matter. As a result, pathogens such as noroviruses, enteroviruses, and *Campylobacter*, which are all known causative agents of gastrointestinal and respiratory diseases, may be detected in floodwater [20,21].

Diarrhea and vomiting are the most commonly reported symptoms of waterborne disease; however, other infections of skin, ear, respiratory tract, or eye are also identified [22]. Flooding of households in Lewes, Southern England was strongly associated with earache in patients of all ages, while weaker associations were observed for skin rash, respiratory illness, and all categories of injury [23]. However, an increase in the risk of gastroenteritis occurrence was significantly associated with depth of flooding [23]. Stomach upsets and recurring flu-like symptoms such as sore throat, cough and general sickness were attributed to the floods and were reported by people whose homes had been affected in Cumbria, northwest England [24].

Direct hand contact with floodwater was found to be significantly associated with increased incidence of gastrointestinal, respiratory, and/or dermatological complaints in the Netherlands and Germany [21,25,26,27]. In parallel, participation in post-flooding cleanup tasks was associated with the development of influenza-like symptoms [27] or both acute gastroenteritis (AGE) and acute respiratory infection (ARI) emergence [21]. Having walked or cycled through floodwater was also related to the development of influenza-like symptoms [27] or AGE occurrence [21].

Waterborne diseases can also be spread while bathing, washing, or eating food exposed to contaminated water. Harder-Lauridsen et al. [28] demonstrated that after an extreme rainfall, physical contact with and unintentional intake of sewage-polluted recreational water can increase the risk of severe gastrointestinal illness. A triathlon sports competition took place in Copenhagen, Denmark shortly after an extreme rainfall in August 2010. The athletes who participated in this event swam in water with high post-flooding bacterial contamination and were found to develop gastrointestinal illness five times more often than athletes who had swum the same distance in unpolluted water [28].

Intense precipitation can transfer pathogenic microorganisms of human or animal fecal origin to the aquatic environment through discharge of raw and treated sewage and run-off from the soil, increasing the microbial load on surface water. A gastroenteritis outbreak occurred in Elassona city, central Greece in March 2012 and was characterized as waterborne because the region had suffered heavy rainfall the week before the gastroenteritis episodes began [29]. Heavy rainfall at the beginning of the month could have contributed to water runoff from fields into rivers, contaminating the water supply with human or animal waste from neighboring dwellings and breeding farms and resulting in increased water turbidity [29]. Tornevi et al. [30] analyzed the relationship between daily fluctuations in gastrointestinal symptoms in Gothenburg population and the amount of rainfall upstream of the drinking water utility exposed to upstream run-offs from agricultural areas and occasionally from overflowing combined sewer systems. It was found that heavy rainfall was associated with an increase in nurse advice calls for gastrointestinal illness on the same day and around 5–6 days later [30].

A matched case-control study in four Nordic countries (Denmark, Finland, Norway, and Sweden) over a 21-year period (1992–2012) was conducted to investigate the association between heavy precipitation events and waterborne outbreaks [31]. Heavy precipitation was found to be positively associated with the occurrence of waterborne outbreaks, especially in spring and summer seasons. It was also noted that groundwater sources as well as single household supplies were particularly vulnerable to extreme weather events [31].

Flooding favored the emergence and incidence increase of waterborne diseases in European countries, as shown in Table S1. Increased incidence or outbreaks of waterborne diseases caused by parasites (*Cryptosporidium* and *Giardia*), viruses (norovirus and hepatitis A virus), and bacteria (*Campylobacter, Escherichia coli, Salmonella, Shigella*) were detected (Table S1) and are thoroughly described below.

#### 3.2.1. Parasites: Cryptosporidium

Heavy rainfall and flooding contributed to an increased risk of *Cryptosporidium* infection in European countries. Confirmed cryptosporidiosis cases were mainly reported in the United Kingdom (UK) [32,33,34,35,36,37] and the Republic of Ireland [38,39,40,41], while only one large cryptosporidiosis outbreak was detected in Germany [42].

*Cryptosporidium* infection may be asymptomatic or develop into diarrhea that spontaneously resolves over a couple of weeks in healthy individuals. On the contrary, immunocompromised patients may experience frequent, life-threatening watery diarrhea that is difficult to treat with currently available medications. Oocysts ingestion, direct contact with infected people or animals, and contaminated water and food are all methods of *Cryptosporidium* fecal-oral transmission. Oocysts of this intestinal parasite can survive in moist soil, water or even harsh environmental conditions for long periods of time. *Cryptosporidium* can also resist conventional disinfection treatments such as chlorination, which increases the risk of water distribution systems contamination [43].

Cryptosporidiosis outbreaks were usually attributed to consumption of unboiled tap water from a specific source [33,36]. Heavy rainfall in the reservoir catchment area supplying raw water to the treatment works contributed to the introduction of *Cryptosporidium* oocysts from the environment into the raw water supply [34,44,45]. The origin of the outbreak was confirmed by the detection of *Cryptosporidium* oocysts in samples from water treatment plants and domestic taps [36].

The spatial distribution of the affected residences in Ayrshire (UK) revealed that most of them shared the same public drinking water supply [32]. A strong statistical correlation has been demonstrated between the reported cryptosporidiosis cases and the residence in an area supplied from two groundwater sources, one of which was found to drain surface water directly from a field carrying cattle feces [35]. In the Republic of Ireland, two cryptosporidiosis outbreaks were reported in April–May 2002 and February–March 2007, and all of the cases were found in regions that used lakes surrounded by farmland as the water source [38,39]. The entry of animal waste into the lake could have been facilitated by heavy rainfall [38,39]. *Cryptosporidium* oocysts were found in the lake’s raw and treated water, as well as in the surrounding environment [38]. A *Cryptosporidium* parvum outbreak was recorded among 35 people (27 pupils and 8 teachers), who took part in a school trip to an outdoor adventure farm in South West England, from May 22 to 26 May 2006. The two most plausible routes of transmission were drinking water from a private well or coming into contact with feces-contaminated surface water following heavy rainfall [37].

Boudou et al. [41] found that the increase in cryptosporidiosis cases across the Republic of Ireland from November 2015 to January 2016 was associated with hydrometeorological variables such as cumulative antecedent rainfall, surface water run-off, and groundwater level [41]. Apart from heavy local rainfall, other environmental factors such as river overflow, shutdown of treatment plants due to mechanical problems, increase in water turbidity, and damage to sewage systems could all contribute to an increased risk of cryptosporidiosis [33,42].

#### 3.2.2. Viruses: Norovirus, Hepatitis A Virus

Human noroviruses (NoVs) represent the leading cause of gastroenteritis outbreaks worldwide, affecting all age groups and being mainly transmitted via an oral-fecal route [46]. NoV is highly contagious due to its low infectious dose and persistence of virus in fecal excretion even weeks after patient recovery [47,48].

NoVs were identified as the main causative agents of the waterborne gastroenteritis epidemics reported in Finland [49,50], France [51,52], Italy [47,51], Greece [53,54], Austria [55], Sweden [56], the Netherlands [57], and Hungary [58].

Acute gastrointestinal disease was detected and related to the microbiological contamination of water supplies following heavy rainfall and flooding events [58]. Floods and surface runoff due to snow melting in spring contributed to groundwater contamination with fecal microbes in Finland and Sweden [49,50,56]. Outbreaks were attributed to drinking non-disinfected ground water [49,50].

According to data from epidemiological and molecular studies, residents living in households connected to the public water network were at a higher risk of developing NoV gastroenteritis [47,56]. In Sicily (Italy), sewage overflowing from septic tanks and latrines as a result of the rainfall caused the contamination with human feces of the wells and springs supplying the public water network [47]. The majority of individuals with symptoms of acute gastroenteritis were drinking water from a single well in Xanthi town, North Eastern Greece [53]. Another large outbreak of non-bacterial gastroenteritis caused by NoVs was reported after heavy rainfall in the same Greek region almost 2 years after the gastroenteritis outbreak described by Papadopoulos et al. [54].

Direct exposure to floodwater contaminated with raw sewage caused the occurrence of a NoV outbreak among American tourists and firefighters who helped pump floodwater out of a hotel inundated due to an extremely heavy rainfall in Salzburg, Austria [55].

Overflow of sewage treatment plants due to heavy rainfall led to the fecal contamination of lakes and rivers in France [51,52]. Consumption of oysters harvested from the Etang de Thau, the second largest lagoon in France, was responsible for NoV gastroenteritis outbreaks not only in France but also in Italy [51]. Oyster consumption following floods near a lagoon with shellfish farming activity was the most important contributing factor in the occurrence of another foodborne viral gastroenteritis outbreak in France [59].

Water contamination by several NoV strains caused an acute gastroenteritis outbreak among swimmers following participation in the Amsterdam City Swim event. Two days before this event, an unusually heavy rainfall caused severe flooding and overflow of the sewage system into the city canals [57].

Hepatitis A virus (HAV) is transmitted through contaminated food and water and thrives in poor sanitary conditions. Increased incidence of HAV infection after flooding was observed in Spain and Italy [60,61]. Apart from extreme rainfall, other climate factors such as weekly day of rainfall and snow were also associated with a rise in the number of Hepatitis A cases in Spain [60].

#### 3.2.3. Bacteria: *Escherichia coli, Campylobacter jejuni, Shigella sonnei*

*Escherichia coli* were the most commonly isolated bacteria followed by *Campylobacter jejuni* and *Shigella sonnei* [19,62,63,64,65,66,67,68].

Shiga toxin or verotoxin-producing *Escherichia coli* (STEC/VTEC) have been related to foodborne and waterborne outbreaks. STEC/VTEC causes a wide range of human gastrointestinal disorders, from watery and bloody diarrhea to hemorrhagic colitis. Infection can also result in the life-threatening hemolytic uremic syndrome (HUS), which is considered to be caused by Shiga toxins (Stx) [69,70]. It is demonstrated that heavy rainfall and high temperature are statistically significantly associated with the occurrence of waterborne VTEC outbreaks [71].

Although cattle are considered to be the primary reservoir of STEC/VTEC strains, sheep are also important carriers of these pathogens [72]. In eastern Scotland, heavy rainfall caused localized flooding on an agricultural showground that was generally used for sheep grazing and hosted a scout camp [62]. *E. coli* O157 infection was detected in 20 campers, and *E. coli* O157 was transmitted from the environment to cases via contaminated hands [62]. In August 2004, seven cases of *E. coli* O157 infection were identified in children on holiday in Cornwall, southwest England [63]. The source of infection was a contaminated freshwater stream flowing across a seaside beach. Because of heavy rainfall in the days preceding the outbreak, feces from cattle found grazing upstream and potentially contaminated by *E. coli* O157 infiltrated the stream, thereby causing the outbreak [63]. Extremely heavy summer rainfall in the Republic of Ireland resulted in high water table levels, intense run-off, and widespread flooding that significantly increased the potential for microbiological contamination of drinking water [64].

During periods of heavy rainfall, areas of stagnogley soils with mixed animal grazing may be more susceptible to *Campylobacter* exposure and spread, increasing the likelihood of human cases of the disease [73]. In Finland, two outbreaks of *Campylobacter jejuni* enteritis were recorded in 2000 and 2001 and were associated to fecal contamination of drinking water sources by surface water runoff after rain [65]. Following an exceptionally heavy rainfall in June 2009, an outbreak of *Campylobacter* gastroenteritis (163 cases) occurred in the Danish town of Tune [66]. Drinking tap water was the only exposure identified as being related with gastroenteritis, with a clear dose–response association between the amount of tap water drunk and the risk of gastroenteritis. Drinking-water contamination was caused by congestion of the combined rainwater drainage and sewage system [66]. Residents of an economically deprived housing estate built on a steep hill and surrounded by agricultural pastures developed *C. jejuni* gastroenteritis after severe rainfall on the surrounding hills in the South Wales Valleys (UK) [67].

An outbreak of acute *Shigella sonnei* gastroenteritis occurred in the town of Santa Maria de Palautordera (Spain), following heavy rainfall that resulted in mud and organic material entering the treatment plants, which were not designed to treat highly turbid water [68].

### 3.3. Rodent-Borne Diseases

**Figure 4:**
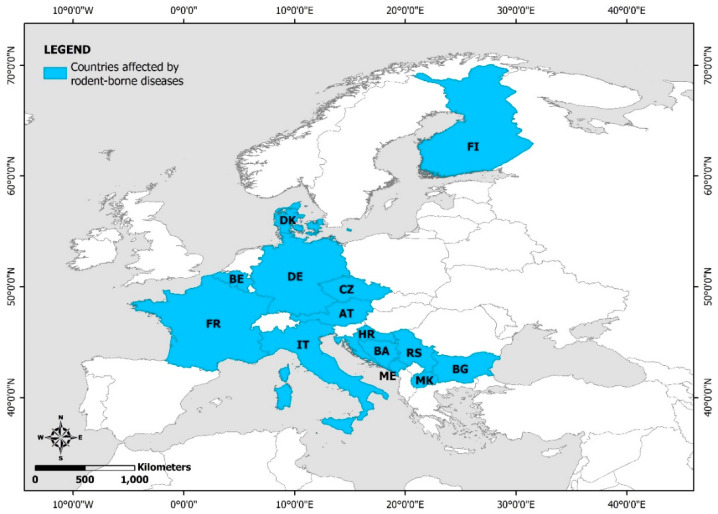
European countries with reported flood-related rodent-borne diseases (FI: Finland; DK: Denmark; FR: France; BE: Belgium; DE: Germany; CZ: the Czech Republic; AT: Austria; IT: Italy; HR: Croatia; BA: Bosnia and Herzegovina; RS: Republic of Serbia; ME: Montenegro; MK: North Macedonia; BG: Bulgaria).

Flood-related rodent-borne diseases have been reported in Finland, Denmark, France, Belgium, Germany, the Czech Republic, Italy, Austria, Croatia, Bosnia and Herzegovina, Montenegro, Serbia, Republic of North Macedonia, and Bulgaria (Figure 4).

#### 3.3.1. Leptospira

Rodent-borne diseases may also increase during heavy rainfall and flooding due to altered patterns of contact among humans, pathogens, and rodents [74]. Numerous outbreaks of leptospirosis, a spirochetal zoonosis, have been associated with extreme weather events and flooding in a wide-range of countries around the world [75,76,77,78,79,80].

Humans can acquire infection through direct contact with infected animal hosts such as rodents, domestic pets, and livestock, or through exposure to surface water or soil contaminated by infected animal urine [81,82]. During floods, the increase in leptospirosis transmission is mainly attributed to closer contact between animal hosts and humans, direct contamination of floodwaters, and damage to water and sanitation networks [83]. There have been reports of flood events associated with sporadic and outbreak cases of Leptospirosis from a wide range of countries in Europe such as Bulgaria [84], the Czech Republic [85], Italy [86,87], Germany [88,89], Austria [90], France [91], and Denmark [92,93,94] (Table S2).

Water-based outdoor sports and recreational activities are becoming increasingly popular. Triathletes, canoers and kayakers, rowers, and wild swimmers have all been known to have acquired leptospirosis infection when participating in outdoor sports and activities [95]. Higher recreational activity, ideal temperature, and rainfall favor *Leptospira* survival in the environment and could plausibly explain the peaks in leptospirosis incidence during the summer months in Bulgaria [84]. Recreational exposure to water, particularly in relation to water sports, is thought to be a key risk factor for leptospirosis occurrence among triathlon athletes in Germany and Austria [89,90]. Heavy rainfall preceding these endurance multisport races is likely to have contributed to the contamination of the man-made lake or the river with *Leptospira* [89,90].

Leptospirosis has traditionally been thought of as an occupational disease, with humans getting infected predominantly through work exposure. Sewage maintenance, animal husbandry, agriculture, mining, and military exercises are activities that increase the risk of contracting leptospirosis [96]. A leptospirosis outbreak was also detected among seasonal harvesters from Eastern Europe, working in the largest field of a strawberry-producing farm in North Rhine-Westphalia, Germany in July 2007 [88]. The warm winter of 2006–2007 enhanced rodent population growth and expansion. The disease risk increased with each day spent working in the rain and the most likely source of the outbreak was direct contact of hand lesions with contaminated water or soil and infected rodents [88]. Work-related cases accounted for nearly half of all leptospirosis cases reported over a 32-year period (1980–2012) in Denmark [92]. Fish farmers, farmers, and sewage workers were the most frequently notified professions [92].

Improper and poor waste management and garbage accumulation attract rat infestation that is related to leptospirosis infection among urban dwellers [96,97]. The emergence of sporadic laboratory-confirmed human cases of leptospirosis in the city of Marseille, France was associated with heavy rainfall periods accompanied by flooding and garbage collection strikes [91]. Garbage left uncollected contributed to expansion of the urban rat population. Residents of Marseille may have been exposed to *Leptospira* through contact with contaminated surface water or rat urine near garbage deposits [91]. High prevalence of pathogenic *Leptospira* spp. in rodents, street flooding, and garbage accumulation could also explain the occurrence of two human leptospirosis cases in the city of Palermo, Italy [87].

Flooding was found to have a significant association with increased incidence of human leptospirosis. When compared to non-flooded areas, widespread flooding may contribute to an increased risk of leptospirosis or create conditions conducive to a leptospirosis outbreak [82]. According to Zitek and Benes [85], the rates of serologically confirmed leptospirosis cases in the Czech Republic were three times higher than usual after the massive floods of 1997 and 2002. Two thirds of these cases came from flooded areas, while half of them were directly exposed to residual water and flood mud in cellars [85]. In August 2002, an excessive rainfall produced a devastating flooding in a suburban area of Vicenza in the northeastern part of Italy. Since only a small percentage of people were wearing personal protective equipment (e.g., gloves and boots) during post-flooding cleanup activities, inundation appeared to be the only demonstrable risk factor for the occurrence of serologically confirmed *Leptospira* infection [86]. Following the 2011 flash floods that left large areas of Copenhagen inundated, a cluster of five leptospirosis cases was detected in Copenhagen [93,94].

#### 3.3.2. Hantavirus

Hantaviruses are carried by different types of rodents, and humans get infected by inhaling aerosolized urine, saliva, or droppings of infected rodent hosts [98]. In Asia and Europe, Old World hantaviruses including Puumala virus, Seoul virus, Dobrava Belgrade virus, and hantaan virus infect the highly differentiated endothelial cells of the kidney, causing acute renal failure with tubular and glomerular involvement, also known as hemorrhagic fever with renal syndrome (HFRS) [98,99]. Globally, 150,000–200,000 cases of hantavirus infection are reported annually, whereas more than 10,000 HFRS cases are diagnosed each year in Europe and their number is growing [98,99].

Heavy rainfall brought on by a cyclone contributed to increases in severe flooding events in the Balkans in mid-May 2014 [100]. HFRS incidence was significantly increased in 2014 in five flood-affected Western Balkan (WB) countries including Bosnia and Herzegovina, Croatia, Montenegro, North Macedonia, and Serbia [101]. A significantly strong negative correlation was found between the monthly incidence of HFRS and the number of months after the May floods for the entire WB area [101].

Higher mean annual precipitations are assumed to reflect higher air humidity and should increase the probability of HFRS occurrence. Zeimes et al. [102] showed a positive association between the risk of HFRS and the annual precipitation in France, Belgium, and Finland, which could be attributed to the good survival of the virus not only within the host but also in a wet environment or to the migration of the rodent population into the human environment in search of food supplies, especially when climatic conditions deteriorate [102].

### 3.4. Vector-Borne Diseases

Vector-borne diseases are infections transmitted by the bite of infected arthropod species, such as mosquitoes, ticks, triatomine bugs, sandflies, and blackflies. Arthropod vectors are especially susceptible to climatic factors, thus weather influences their survival and reproduction rates. The geographical distribution of vector-borne diseases is determined by the geographical distribution of both vertebrate host and vector [83]. Consequently, the risk of vector-borne diseases could increase if vector-borne pathogens are present along with their competent vectors in the flooded areas.

Precipitation changes are known to affect the reproduction; development; and behavior of arthropod vectors, their pathogens, and non-human vertebrate reservoirs [55]. Floods may indirectly increase the incidence of vector-borne diseases through the expansion in the number and range of vector habitats. Flood waters initially overwhelm breeding habitats and temporarily wash out mosquito populations. However, receding water could provide ideal mosquito breeding grounds and, therefore, enhance the potential for exposure of the flood-affected population and emergency responders to mosquito-borne pathogens causing diseases such as West Nile fever, malaria, and dengue fever [103].

In a 6-year investigation (2011–2016), three zoonotic arboviruses comprising of Usutu, Sindbis, and Batai viruses were found circulating in the German mosquito fauna. According to Scheuch et al., the Elbe flood could explain the relatively high number of pathogen findings in 2013, as flood events favor the mass development of high numbers of mosquitoes, which in turn facilitate enhanced virus circulation [104].

**Figure 5:**
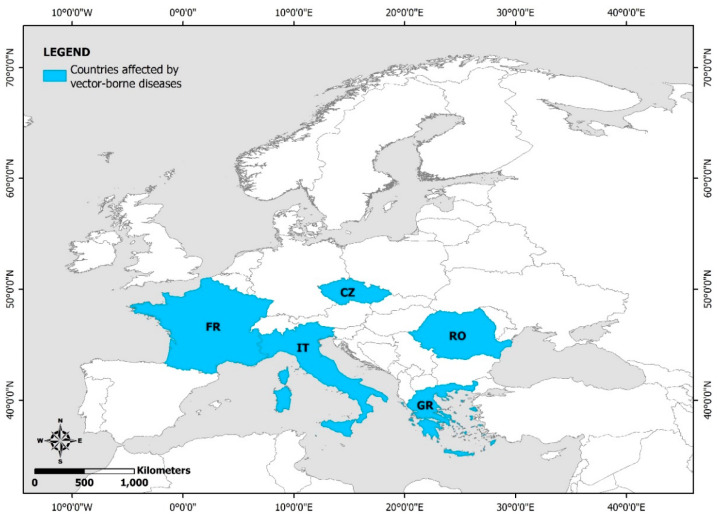
European countries with reported flood-related vector-borne diseases (FR: France; CZ: the Czech Republic; RO: Romania; IT: Italy; GR: Greece).

Outbreaks of mosquito-borne diseases associated with heavy rainfall or floods have mainly been observed in tropical areas [103,105]. In Europe, flooding events following extreme rainfall have been mainly linked to the emergence and incidence increase of West Nile virus (WNV), Chikungunya virus (CHIKV), and Tahyna virus (TAHV) infections in Romania, the Czech Republic, Greece, Italy, and France (Figure 5; Table S3) [106,107,108,109,110,111,112].

In the summer of 1996, an unprecedented epidemic of WNV meningoencephalitis (393 hospitalized cases and 17 deaths) occurred in southeastern Romania. Han et al. [106] found WNV infection to be associated with specific residence characteristics, such as presence of mosquitoes indoors and flooded basements of apartment buildings. It is worth mentioning that the basements were inundated with sewage-contaminated water from poorly maintained plumbing, resulting in a high-organic environment conducive to mosquito breeding [106].

After heavy rainfall in Moravia (Czech Republic), the devastating flooding of the Morava River occurred in July 1997. In the flood-affected area, the abrupt increase of mosquitoes infected with arboviruses such as WNV and TAHV contributed to the detection of confirmed and probable cases of WNV infection in that area [107]. Climatic parameters such as high temperature and increased precipitation might have a lagged direct effect on the incidence of WNV infection in Northern Italy [112].

In August 2002, a massive flood struck Prague, the capital of the Czech Republic, as well as extensive rural areas along the Vltava and Labe Rivers. An elevated incidence of “Valtice fever” caused by TAHV was found among inhabitants of the flood-affected areas in Central Bohemia, while WNV, Sindbis virus, and Batai virus infections were not reported. The prevalence of antibodies against TAHV increased with decreasing distance from areas with high mosquito abundance and floodplain forests, the primary mosquito breeding habitats [108,109].

Roiz et al. carried out surveillance of the Asian tiger mosquito *Aedes albopictus*, a well-known CHIKV vector in Montpellier (France) and found that extreme rainfall that inundated the city in 2014 clearly contributed to an increase of mosquito population growth and abundance, as well as to the prolongation of the autochthonous CHIKV transmission period [111].

An outbreak of WNV infection occurred in the Central Macedonia, (northern Greece) in the summer of 2010. A total of 197 patients with neuroinvasive disease were reported, of whom 33 (17%) died [110]. Danis et al. (2011) noticed that the 2010 WNV outbreak was preceded by unusual precipitation. According to meteorological data for the area, 2010 was warmer than previous years and unusually wet [113].

## 4. Strategies and Measures for Prevention and Management of Infectious Disease Outbreaks in Flood-Prone and Flood-Affected Areas Respectively

### 4.1. Preventing an Infectious Disease Outbreak

The likelihood of infectious disease outbreaks following flooding is associated with the regional disease incidence, the type and extent of the event, the resilience of public health infrastructure, and the effectiveness of the disaster response [114]. Disease surveillance and early warning systems, coupled with effective prevention and response capabilities, can reduce current and future vulnerability to infectious diseases following floods.

Waterborne infectious diseases are a major public health issue in developing countries [115]. However events that occurred in the aforementioned European countries show that waterborne outbreaks can also occur in developed countries and may have a significant negative impact on public health. Floods in high-income countries are regarded to be rare in terms of causing infectious disease epidemics, and when they do, they are thought to be readily handled and limited in scope due to the rapid implementation of preventive measures.

The first important step for preventing an infectious disease outbreak is its identification and the clarification of their most important risk factors [116]. Taking into account Mavrouli et al. [117] and Mavroulis et al. [118], the risk factors for emergence and incidence increase of the most fatal post-flood infectious diseases described worldwide comprise: (1) poor economic status and living in flood prone areas; (2) destruction of infrastructures, disruption of public utilities, and interruption of basic public health services; (3) direct physical exposure to sewage-polluted flood water; (4) lack of adequate potable water and water-supply from contaminated ponds and tube wells along with lack of distribution of water purification tablets; (5) aggravation of environmental conditions comprising rapid cooling of the environment and heightened humidity; (6) population displacement resulting in densely populated and overcrowded regions; (7) unfavorable living conditions in emergency shelters; (8) improper and inadequate sanitation or no access to clean water and sanitation; (9) proliferation and abrupt increase of vector and rodent populations after flooding; and (10) contamination of water, damp soil, mud or vegetation caused by rodent urine, dead animals, and overflow of latrines.

By giving particular emphasis on the aforementioned risk factors, the preparedness for natural disasters constitutes a critical and urgent need and should comprise several actions at all administration levels (central, regional, and local). Education, training, and awareness-raising activities for the identification and management of infectious diseases may contribute to the reinforcement of the health-surveillance systems and the maintenance and delivery of effective and efficient health care services aiming at the reduction of the related mortality and morbidity [116].

The awareness-raising and information actions in flood-prone areas could be useful in avoiding the onset of an infectious disease outbreak before, during or after flooding, or in mitigating and eliminating the adverse effects on the public health after the onset of an infectious disease outbreak. These actions allow for the successful management of an upcoming outbreak, as well as the easy implementation of personal protective measures to avoid infection.

Based on recent flooding in Europe including events in 2007 in Germany [88], in 2011 in Copenhagen [26], and in 2013 in the Netherlands and Germany [27,42], the competent authorities should advise professionals and residents to wear personal protective equipment (PPE) to avoid contact with floodwater and water bodies that may be contaminated as a result of flooding, and to practice proper hand hygiene in order to prevent initial infection and further transmission within the flood-affected communities.

In order to prevent an outbreak of leptospirosis, for example, information should be provided to the general public and disaster management officials about how to avoid contact with water, especially if skin abrasion occurs; how to avoid touching eyes, nose, and mouth since *Leptospira* can enter through mucous membranes; how to recognize the symptoms of leptospirosis infection; and how to seek immediate medical attention and advice if fever or illness occurs [86]. Leptospirosis is frequently overlooked as a cause of fever or systemic illness. Its diverse symptoms and non-specific presentations can be mistaken for a variety of other infectious diseases [83,119]. Early identification of signs and symptoms, proper treatment, and implementation of disease-specific therapies are critical to reduce morbidity and mortality, since misdiagnosis or delayed diagnosis has serious clinical implications. Therefore, clinicians should consider leptospirosis as a differential diagnosis for febrile infections following floods [119].

Staying on the floodplain, as well as swimming and playing in water bodies such as lakes, rivers, and pools that may be contaminated and have increased water turbidity during flooding, should be avoided [42,90,113]. According to the findings of Socolovschi et al. [91], children should avoid playing in yards, rain puddles, or mud probably contaminated with urine of infected animals commonly including rats, mice, cows, pigs, and dogs.

Residents should avoid garbage deposits where they could be exposed to contaminated surface water or urine of infected animals [91]. Following floods, everyone exposed to surroundings with a high risk of leptospirosis should get time-bound chemoprophylaxis to reduce the leptospirosis incidence, as well as related morbidity and mortality during outbreaks [120,121]. In an endemic area, however, leptospiral infection may not be effectively prevented [89,90,122,123].

Applying personal protective measures is critical for the health of the affected local population and the staff involved in the emergency response and recovery actions, including first responders; members of search and rescue (SAR) teams; staff of the cleaning services; and volunteers, among others, during the first hours and days after flood onset. Nowadays, because of the evolving COVID-19 pandemic and the continuous effort to mitigate the adverse effects on humanity, most of these measures have become widely known and include:

- regular hand washing with soap and clean, safe running water, or alternatively disinfecting hands with alcohol-based wipes or hand sanitizers that contain at least 60% alcohol in case of lack of soap and water, especially after contact with floodwater and before eating; drinking; touching eyes, nose, and mouth; and treating a cut or wound;
- wearing PPE comprising disposable gloves, rubber boots, and waders for protecting body and limbs from contact with floodwater, related sediments, and debris; and
- wearing face protection gear including mask and safety goggles and glasses for protecting against splashes and droplets in eyes, nose, and mouth.

It is important to note that hands can be contaminated through removing the PPE. Residents wearing PPE, such as disposable gloves, often experience a false sense of safety and do not apply proper hand hygiene measures after their removal. Furthermore, some parts of the equipment could form ideal conditions for new infections. Disposable gloves, for example, may develop holes and tears that are not immediately detected, resulting in optimum moisture and temperature for rapid bacterial proliferation and infection emergence. Masks, boots, and waders may be contaminated and should be carefully removed and disposed of (for disposable parts) or cleaned up and stored properly (for non-disposal parts).

According to the recommendations of the Centers for Disease Control and Prevention [113]), all clothes used during the cleanup process or other actions in the flood-affected area that require contact with flood waters, sediments, and debris should be washed separately from uncontaminated clothes in hot water and detergent.

### 4.2. Post-Flood Measures for Preventing an Infectious Disease Outbreak

As regards the post-flood measures for the prevention of infectious disease outbreaks, the disinfection of the inundated buildings should follow the cleaning-up processes in the flood-affected areas. All household equipment, including furniture and electric appliances, as well as non-structural elements comprising wooden floors, doors, and windows that cannot be disinfected, should be removed. Mechanical disinfection should follow with the removal of mud and dirt from surfaces such as walls, ceilings, and floors. Chemical disinfection should complete the process with motor-spraying surfaces with chlorine-based disinfectants. These steps were followed by residents in Croatian villages after the floods in 2014 in order to prevent and control communicable diseases in the post-disaster period [124].

Within the first week of the flooding, a rapid risk assessment should be conducted by recording information on the flood-affected area and population with special emphasis on displaced people, as well as the on the risk of infectious disease outbreaks and disruption of public health infrastructure [7,125]. Recorded information contributes to the establishment of adequate disease surveillance systems and the identification of appropriate interventions for managing and mitigating the adverse effects of infectious disease outbreaks that occur simultaneously or subsequently to a flood disaster [7,125,126,127,128]. Since disasters induced by natural hazards do not import new diseases to the affected areas, an infectious agent can be transmitted if it is endemic to the affected region or if it is introduced into that region through SAR operations [129]. Thus, when environmental conditions become favorable, infectious diseases already endemic in a particular area may grow into an outbreak [129].

Public health authorities should prioritize the surveillance of vector-borne diseases, including those transmitted by mosquitoes, as well as of rodent-borne diseases to minimize the risk of mosquito- and rodent-borne diseases across Europe. The recognition and identification of local vector and rodent species, environmental factors, and breeding habitats that influence local disease transmission is important for the implementation of response activities and control measures and the improvement of preparedness activities aimed at emerging mosquito- and rodent-borne diseases.

Mosquito and rodent control measures were implemented in the eastern part of Croatia after the 2014 floods to prevent infectious disease transmission [124]. They were also recommended for the areas in Bosnia and Herzegovina, Croatia, and Serbia affected by floods in the same year [130]. An integrated vector management program comprised larval control applied in the pre-outbreak phase in all flood-affected areas, adult mosquito control in order to reduce the number of infected female mosquitoes, reduction of mosquitoes breeding sites, and of the reproduction capacity of the local vector populations as well as vector surveillance [130].

Stagnant water serving as a common breeding habitat for mosquitoes should be regularly checked, while rodenticides were appropriately applied in all households in the flood-affected and high risk areas [124]. Apart from regular checking, stagnant water should be removed where possible. For instance, water storage containers should not be left unattended and become suitable aquatic breeding sites for female mosquitoes to lay their eggs. Residents could also use thick clothing, insect and pest repellents, insecticide vaporizers, mosquito nets, and door and window covers as personal protective measures.

Furthermore, the local rodent species and their behavioral characteristics should be recognized by evaluating all available sources. Supplies storage sites and households close to the flood-affected area should be protected by rodent attacks. Areas and facilities, including local landfills, in which conditions may be conducive to rodent population growth, should be better monitored, and waste should be properly and safely stored and disposed [18]. These actions have high potential for reduction of rodent population and subsequently for prevention of rodent-borne disease emergence and incidence increase [131].

Due to the ongoing climate crisis, more frequent extreme weather events could exacerbate infectious disease transmission and spread by displacing populations, damaging public health and sanitation infrastructure, and disrupting routine public health activities, including vaccination [132]. In addition to the aforementioned measures for preventing and controlling infectious diseases after flood occurrence, vaccination programs could also be implemented in susceptible and vulnerable areas [116,125]. These programs constitute the most cost-effective interventions in public health and an immediate health priority for decreasing the microbiological load of infectious agents directly among the local affected population. This is essential for the case of evacuation due to floods and population displacement into temporary, overcrowded accommodation and among the staff of authorities and services, contributing to the response and recovery process. In addition to COVID-19, an increased risk of transmission of vaccine-preventable diseases including measles, meningitis, varicella, and influenza could be observed when displaced people are temporarily housed in close proximity in overcrowded shelters [133]. However, the risk is higher in countries with inadequate vaccination coverage and limited resources [134].

### 4.3. Mitigation Actions When an Infectious Disease Outbreak Occurs

When an infectious disease outbreak occurs, the preventive measures give way to mitigation actions. In case of a waterborne disease outbreak after flooding, households could adopt protective measures comprising immediate discontinuation of contaminated water use, boiling of drinking water, or utilizing different potable water sources in order to mitigate the adverse effects of the outbreak [50]. As regards the effective disinfection of the public water works, shock chlorination and flushing of water pipelines could be applied to eliminate the pathogens attached in the pipelines [50].

Such mitigation measures were applied in the city of Halle (eastern Germany), after the early June 2013 extreme river flooding attributed to several days of heavy rain and Saale River banks overflowing. The competent authorities provided information via press release about an outbreak of *Cryptosporidium hominis* and initially advised people to boil drinking water and to avoid swimming, sunbathing, and playing close to water bodies comprising rivers and pools probably contaminated by flooding [42]. Regular sampling and testing of water bodies were also conducted [42].

As regards post-flood mitigation actions in higher levels of administration, the local and regional authorities and the central government could implement several actions for responding to the emergency flood situation and controlling infectious disease outbreaks. These actions mainly comprise first-aid treatment and medical care provided during the implementation or after the completion of SAR operations in residents having signs and symptoms of disease; provision of emergency shelters for evacuees, homeless, and those in need after flooding; and distribution of essential emergency supplies. The continuous provision of the adequate clean and safe water for drinking and cooking as well as the preservation of water and food quality until consumption constitute the most important measure for controlling waterborne and fecal-oral diseases in a flood-affected area and mitigating their effects. It is found that proper hand washing can reduce diarrhea episodes by one-third [135].

As regards the long-term prevention, there is need for flexible preparedness planning and measures with ability for quick and effective integration of unforeseen conditions and related adverse effects [136]. This approach requires the improvement of our knowledge on the relation between infectious diseases and disasters induced by hydrometeorogical hazards. Measures for preventing infectious disease outbreaks and mitigating their effects on the affected population and the involved staff should be incorporated into the emergency response planning for disasters induced by natural hazards [137]. Furthermore, these measures should be timely and effectively communicated not only to the staff involved to the prevention and management of these emergencies but also to the general public and in particular to residents belonging to certain groups within communities, which may be more vulnerable than others to the adverse effects of flood events [137], and therefore may suffer the most by the generation of such events and subsequent infectious diseases’ outbreaks. These groups comprise the elderly population, persons with disabilities, children, women, low-income individuals, homeless, and migrants [138].

### 4.4. When Flooding, Flood-Related Infectious Disease Outbreaks and COVID-19 Collide

As can be seen from the above, the management of the flood impacts and possible associated infectious disease outbreaks has already been a complex issue and a typical example of multi-hazard management that requires a truly interdisciplinary approach. This issue has become even more complex during the evolving COVID-19 pandemic as many relevant studies and cases around the world show that conflicting issues emerge between response actions and pandemic mitigation measures [139,140,141,142,143,144,145,146]. For example, emergency actions, such as SAR operations among others, require close and frequent contact not only with the flood-affected local population and authorities, but also with other professionals, rescue teams and volunteers from different areas with different epidemiological characteristics. Additionally, thousands of evacuees should be accommodated in emergency shelters, resulting in overcrowding and high mobility in these sites. As regards the emergency supplies, many volunteers participate in the entire process from the collection and the preparation of emergency supplies to their final distribution to the affected population. Furthermore, many people including evacuees; responders; and volunteers share areas, surfaces, items, and equipment not only in the emergency shelters but also in several other sites of the affected area. These actions could pose higher risk for disease transmission within the affected community and have the potential to create SARS-CoV-2 clusters within the affected area, which will affect emergency response and slow down the recovery process [146].

The impact of disasters on the COVID-19 pandemic evolution in an affected area during the post-disaster period may include local increasing, decreasing, or stability of the COVID-19 cases. These trends depend on several factors, which deal with the epidemiological characteristics of the virus in the affected area, the demographic characteristics of the affected area, as well as the magnitude and the intensity of the generated disaster [144]. More specifically, they are related to:

- the pre-existing community prevalence of COVID-19 in the disaster affected area;
- the number of people involved in assessing disaster impact and, in response, actions in the disaster affected area;
- the measures applied by responders for personal protection and for the safety of the disaster-affected population especially during the first crucial days of the emergency response;
- the intensity of the generated events and their effects on population (fatalities and injuries), on nature (environmental effects) and on the building stock (structural damage to buildings, infrastructures, and lifelines);
- the immediate housing measures adapted to the unprecedented conditions;
- the need for immediate evacuation without the contribution and support of the Civil Protection staff, responders, and volunteers;
- the accessibility to the affected area during the pre- and post-disaster periods;
- the demographic properties of the affected area with emphasis on the population density and the geographical distribution of residential areas; and
- the level of education, organization, and preparedness of the authorities, agencies, and services involved in the management of the simultaneous impacts of natural hazards and related disasters, as well as biological hazards, such as pandemics and outbreaks.

Whether responding to floods or a pandemic, an effective approach is required. Countries should achieve sustainable development in order to address these issues. As the parallel occurrence of hydrometeorological hazards, infectious diseases, and the evolving COVID-19 pandemic has shown, multihazard conditions have a high potential to amplify related disasters. In order to reduce this potential, a multihazard approach in disaster management and disaster risk reduction is required, with the following actions being the most important, as recommended by literature reviews and expert panels (e.g., [144,145,146,147]):

- Improving whole-of-society coordination mechanisms to aid preparedness, including health, transportation, travel, security, and other crisis first responders.
- Setting up emergency camps or makeshift settlements for displaced people forced to flee their homes.
- Increasing the number of emergency shelters of the same type or using other facilities as emergency shelters in order to avoid overcrowding and maintain physical distancing.
- Configuring individual rooms, separate areas, large facilities, and guidelines for maintaining physical distancing within the emergency shelters.
- Modifying food preparation and distribution practices and adapting to the unprecedented conditions. For instance, packaged meals could be prepared and served on individual and disposable serving utensils by staff wearing masks and disposable gloves with working surfaces being cleaned and disinfected on a regular basis.
- Establishing a surveillance system in emergency shelters and temporary settlements to detect COVID-19 patients early in the displaced population and isolate sick individuals including temporary isolation facilities equipped with the appropriate medical-technological equipment for health assessment, medical care, and counseling.
- Establishing health-care centers or field hospitals for the control of critical points for managing COVID-19-infected waste.
- Meeting the flood-affected people’s immediate health needs, with emphasis on vulnerable people, such as the elderly, women, and children.
- Intensive training and reminders about public health measures such as hand hygiene, respiratory etiquette, and social distancing to health-care providers in provinces that have received flood warnings. Medicine supply, distribution location determination, and patient access to outpatient drugs, as well as delivery and drug distribution personnel training.
- Weekly distribution of sufficient quantities of hygiene items comprising masks, soap, and waterless antiseptic agents including alcohol-based solutions for hands and surfaces disinfection among flood victims.
- Construction of control shared bathrooms, use of clean surfaces with water and detergents, and access to appropriately designed toilets to prevent contamination of groundwater resources.
- Access to psychological consultations and support for flood-affected people, especially if they have lost a family member due to COVID-19.

Prior to and during the occurrence of floods, policymakers and healthcare managers in flood-prone areas should emphasize the above measures. It is critical for policy makers to encourage stakeholders to use the managerial considerations suggested in this study to manage floods, flood-related outbreaks, and COVID-19.

## 5. Conclusions

Disaster risk reduction and disaster management since early 2020 has faced many challenges. These challenges arise when different types of hazards occur simultaneously or evolve in parallel. A typical recent example is the occurrence of destructive floods in Central Europe countries in the summer of 2021, in the midst of the evolving COVID-19 pandemic, with significant population impacts (human casualties, injured and homeless) as well as impacts on the natural environment (flooding of extensive areas, erosion, and deposition in places) and the built environment (severe structural damage especially to structures and infrastructure close to overflowed rivers and streams). The widespread and severe impacts created harsh conditions, ideal, as the related literature suggests, for the onset of post-flood infectious diseases.

Considering this high potential for causing infectious diseases, a systematic literature review was conducted in order to see if similar events of parallel hydrometeorological and biological health hazards have occurred in the past and if there are aggravating risk factors. It was found that the hazard really exists as in many European countries conditions have been created by the occurrence of mainly rainfall and subsequent flooding, leading to the occurrence of water-borne, rodent-borne, and vector-borne infections, and related outbreaks, as shown above.

With the climate crisis now evident and evolving, hydrometeorological hazards and related disasters are already on an upward trend, which is expected to increase even more in the upcoming years (e.g., [148,149,150,151,152,153,154,155,156,157,158]), greatly affecting or sometimes even suspending activities in many sectors of daily life. This increase in hydrometeorological hazards and related disasters affects the interaction between lithosphere, hydrosphere, and biosphere, which in turn can form multi-hazard conditions with the simultaneous occurrence or parallel evolution of hydrometeorological hazards including floods and biological health hazards including infectious disease outbreaks.

Effective management of multi-hazard conditions requires interdisciplinary and multi-hazard approaches, which often combine earth, medical, and natural hazards sciences and can ensure a more effective and immediate response as well as a faster recovery.

These approaches include public health interventions to prevent the health impacts of floods and in particular to reduce vulnerability to infectious diseases, including enhanced post-flood epidemiological surveillance systems, risk assessments, and specific prevention and control strategies, depending on the characteristics and properties of the generated events. The current COVID-19 pandemic underlined the importance of a common effort and approach to ensure safety and health across Europe. In addition, the 2021 recent flood disasters in European countries called for immediate joint action, in this case regarding the prevention and management of water-, rodent- and vector-borne infectious diseases.

## Data Availability

Not applicable.

## Supplementary Materials

The following supporting information can be downloaded at: https://www.mdpi.com/article/10.3390/ijerph191610206/s1. Table S1. Waterborne diseases related to hydrometeorological hazards in Europe; Table S2. Rodent-borne diseases related to hydrometeorological hazards in Europe; Table S3. Vector-borne diseases related to hydrometeorological hazards in Europe.
