# Supplementary material for: Infectious Diseases Associated with Hydrometeorological Hazards in Europe: Disaster Risk Reduction in the Context of the Climate Crisis and the Ongoing COVID-19 Pandemic
Source: Int J Environ Res Public Health. 2022 Aug 17;19(16):10206. doi: 10.3390/ijerph191610206 (PMC9408126; doi:10.3390/ijerph191610206)
Supplement: Supplementary file 1 [file ijerph-19-10206-s001.zip › ijerph-1823266-supplementary/Table_S1_Waterborne diseases.pdf]

Table S1. Waterborne diseases related to hydrometeorological hazards in Europe.

Country	Area	Infectious disease(s)	Causative pathogens	Infectious disease(s) occurrence	Hazard type	Refs.
UK	Ayrshire (SW Scotland)	Cryptosporidiosis (27 cases)	Cryptosporidium	24.03.1988-23.04.1988	Heavy rainfall	[32,44,45]
UK	Isle of Thanet (Kent, England)	Cryptosporidiosis (47 cases)	Cryptosporidium	12.1990-02.1991	Heavy rainfall	[33]
UK	Bradford (W. Yorkshire, England)	Cryptosporidiosis (125 cases)	Cryptosporidium	11-12.1992	Heavy rainfall	[34]
UK	Warrington (North West England)	Cryptosporidiosis (47 cases)	Cryptosporidium	11.1992-02.1993	Excessive rainfall	[35]
FI	Heinävesi (Finnish municipality)	An epidemic of acute gastroenteritis	Norwalk virus<br>Adenovirus<br>Small round viruses<br>Group A and C rotaviruses	04.1994	Flood	[49]
FI	Several communities	14 waterborne epidemics	Norwalk-like viruses (8 out of 14 epidemics)<br>Campylobacter sp.(3 out of 14)	02-05 (13 out of 14) 06-07 (1 out of 14), 1998-1999	Floods<br>Surface runoffs	[50]
UK	Several areas (England and Wales)	89 drinking water-related outbreaks	Giardia<br>Cryptosporidium<br>Escherichia coli<br>Salmonella typhi<br>Salmonella paratyphi<br>Campylobacter	In various periods from 1910 to 1999	Heavy rainfall	[19]

			Streptobacillusmoniliformis			
UK	Clitheroe (Lancashire, England)	Cryptosporidiosis (58 cases)	Cryptosporidium sp.	03.2000	Heavy rainfall	[36]
UK	New Deer (Aberdeenshire, Scotland)	Gastroenteritis (20 cases)	Escherichia coli O157	05.2000	Heavy rainfall	[62]
FI	1st: community in S. Finland<br>2nd: community in E. Finland<br>3rd: community in S. Finland	3 outbreaks of gastroenteritis:<br>1st: 400 cases<br>2nd: 50 cases<br>3rd: 1000 cases	Campylobacter jejuni	1st: 07-08.2000<br>2nd: 08.2001<br>3rd: 10-11.2001	Heavy rainfall	[65]
UK	South Wales	Gastroenteritis (15 cases)	Campylobacter jejuni	09.2000	Heavy rainfall	[67]
UK	East Sussex, Lewes (England)	Gastroenteritis<br>Ear, skin and respiratory infections	n/a	10.2000	Severe river flooding	[23]
IT	Piemonte<br>Lombardia<br>Liguria	Hepatitis A<br>Legionellosis<br>Infectious diarrhea	Hepatitis A virus<br>Legionella pneumophila	In various periods from 2000 to 2002	Floods	[61]
IE	Midland Health Board	Cryptosporidiosis (29 cases)	Cryprosporidium	04-05.2002	Heavy rainfall	[38]
DE	Saxony	Diarrhea	n/a	08.2002	Flood	[25]
ES	Barcelona	Gastroenteritis (181 cases)	Shigella	08-09.2002	Heavy rainfall	[68]
FR<br>IT	Etang de Thau Lagoon (FR)<br>Meze site (IT)	Gastroenteritis<br>Paris: 21 cases (19 and 24 December 2002)	Noroviruses	12.2002	Heavy rainfall<br>Floods	[51]

		Hérault, Ile de France, Aude and Côte d'Or (FR): 69 cases (14 and 25 December 2002)<br>IT: 200 cases				
UK	Cornwall (SW England)	acute gastroenteritis	Escherichia coli O157	08.2004	Heavy rainfall	[63]
GR	Xanthi	Gastroenteritis (709 cases)	Norovirus	28.01.2005<br>10.02.2005	Heavy rainfall<br>Subsequent flood	[53]
UK	Carlisle (Cumbria, England)	Stomach upsets<br>Recurring flu-like symptoms (sore throat, cough and general sickness)	n/a	01.2005	Floods	[24]
AT	Salzburg	Gastroenteritis (16 cases)	Norovirus	05-06.2005	Flood	[55]
FR	A shellfish production lagoon	Gastroenteritis (205 cases)	Enteric viruses	02.2006	Flood	[59]
UK	Cornwall (SW England)	Cryptosporidiosis (35 cases)	Cryptosporidium	22-26.05.2006	Heavy rainfall	[37]
HU	Miskolc	Gastroenteritis (3,673 cases)	Campylobacter<br>Noroviruses	03.06-22.06.2006	Heavy rainfall<br>Subsequent flood	[58]
GR	Xanthi	Gastroenteritis (1,640 cases)	Norovirus	05.06-03.09.2006	Poor hygienic conditions<br>Heavy rainfall	[54]
FR	Several areas	Acute gastroenteritis (10 outbreaks)	Noroviruses<br>Cryptosporidium sp.	In various periods from 1998 to 2006	Heavy rainfall leading to surface runoff and river overflow	[52]

IE	Galway city and county	Cryptosporidiosis (182 cases)	Cryptosporidium	02-03.2007	Rainfall<br>Lake water contamination	[39]
IE	Cork and Kerrycounties	Waterborne disease (148 cases)	Vero cytotoxin-producing Escherichia coli (VTEC)	08.2008	Exceptionally heavy rainfall leading to unprecedented high water table levels, marked runoff and extensive flooding	[64]
SE	Mountain village	Gastroenteritis (173 cases)	Noroviruses	04.2009	Considerable amounts of snow melting (overflow of the drinking water supply network)	[56]
DK	Tune town	Gastroenteritis (163 cases)	Campylobacter	06.2009	Heavy rainfall	[66]
DK	Copenhagen	Gastroenteritis (351 cases)	Campylobacter<br>Giardia lamblia<br>Diarrheagenic E. coli	08.2010	Heavy rainfall	[28]
IT	Sicily	Gastroenteritis (156 cases)	Noroviruses	02-03.2011	Heavy rainfall	[47]
DK	Copenhagen	Diarrhea<br>Cold/sore throat<br>Headache<br>(56 cases among employees engaged in	n/a	02.07-25.07.2011	Flood	[26]

		post-flood management activities)				
SE	Gothenburg	Gastroenteritis	n/a	11.2007-12.2011	Heavy rainfall	[30]
GR	Elassona	Gastroenteritis (552 cases)	Rotaviruses	03.2012	Heavy rainfall	[29]
DK<br>FI<br>NO<br>SE	Several areas	Waterborne disease	n/a	Spring-Summer Autumn-Winter, from 1992 to 2012	Heavy precipitation	[31]
IE	Several areas	Waterborne disease (32 outbreaks, 137 confirmed cases)	Vero cytotoxin-producing Escherichia coli (VTEC)	In several periods from 2005 to 2012	Heavy rainfall	[71]
DE	Halle	Cryptosporidiosis (1 outbreak, 167 cases)	Cryptosporidium	08.2013	Heavy rainfall leading to river overflow	[42]
NL	7 flooded locations presented in a map	Gastrointestinal, dermatological and Influenza-like illness	n/a	06-07.2013	Heavy rainfall<br>Flood	[27]
ES	Several areas	Hepatitis A	Hepatitis A virus	2010-2014	Heavy rainfall<br>Snow	[60]
NL	60 locations	Acute gastroenteritis<br>Acute respiratory infection	n/a	Summer 2015	Flood	[21]
NL	Amsterdam	Acute gastroenteritis	Noroviruses	09.2015	Heavy rainfall leading to severe flooding causing	[57]

					sewage overflow into the city canals	
IE	Several areas presented in a map	Enteritis<br>Cryptosporidiosis	Verotoxigenic E. coli (VTEC)<br>Cryptosporidium	04.2016	Storms<br>Fluvial and pluvial flooding	[41]
