# Supplementary material for: Infectious Diseases Associated with Hydrometeorological Hazards in Europe: Disaster Risk Reduction in the Context of the Climate Crisis and the Ongoing COVID-19 Pandemic
Source: Int J Environ Res Public Health. 2022 Aug 17;19(16):10206. doi: 10.3390/ijerph191610206 (PMC9408126; doi:10.3390/ijerph191610206)
Supplement: Supplementary file 1 [file ijerph-19-10206-s001.zip › ijerph-1823266-supplementary/Table_S2_Rodent-borne diseases.pdf]

Table S2. Rodent-borne diseases related to hydrometeorological hazards in Europe.

Country	Area	Infectious disease(s)	Cases	Infectious disease(s) occurrence	Hazard type	Refs.
BU	n/a	Leptospirosis	455	1989-2001, late summer and early autumn with a peak incidence in August	Rainfall	[84]
IT	Vicenza	Leptospirosis	1 fatal case of leptospiral haemorrhagic pneumonia<br>7 of the 44 people screened: anti-Leptospira IgM antibodies<br>3 cases: rising of antibody titers after re-testing in December 2002	August 2002	Flood	[86]
CZ	n/a	Leptospirosis	1997: 94 individuals (1.3 % of people tested)<br>2002: 92 individuals (1.8 % of people tested)	07.1997<br>08.2002	Flood	[85]
DE	Heidelberg and on the Neckar river	Leptospirosis	5 confirmed cases (1st leptospirosis outbreak related to a competitive sports event in Germany)	08.2006	Heavy rainfall	[89]

DE	Duren in North Rhine-Westphalia	Leptospirosis	13 confirmed cases<br>11 suspected cases	06-07.2007	Heavy rainfall (08-11.06.2007)	[88]
IT	Palermo	Leptospirosis	2 cases	Spring and fall 2009	Storms (20.04.2009)Heavy rainfall (16.09.2009 21.09.2009 25.09.2009 13.10.2009)	[87]
AT	Langau village (Lower Austria)	Leptospirosis	4 serologically confirmed cases (1st leptospirosis outbreak reported in Austria)	07.2010	Heavy rainfall (18-19.06.2010)	[90]
FR	Marseille	Leptospirosis	3 human cases	10.2009-02.2010	Heavy rainfall with flooding (09-10.2009)	[91]
DK	Copenhagen	Leptospirosis	5 cases	07.2011	Rainstorm 02.07.2011	[93,94]
DK	n/a	Leptospirosis	584 laboratory-confirmed leptospirosis cases	In several periods from 1980 to 2012	n/a	[92]
BA<br>HR<br>ME<br>MK<br>RS	n/a	Hemorrhagic fever with renal syndrome	1065 patients were reported in five Western Balkan countries	2014	Heavy rainfall (05.2014)	[101]
FR<br>BE	n/a	Hemorrhagic fever with renal syndrome	n/a	n/a	Higher mean annual precipitations	[102]

FI						
