# Supplementary material for: Infectious Diseases Associated with Hydrometeorological Hazards in Europe: Disaster Risk Reduction in the Context of the Climate Crisis and the Ongoing COVID-19 Pandemic
Source: Int J Environ Res Public Health. 2022 Aug 17;19(16):10206. doi: 10.3390/ijerph191610206 (PMC9408126; doi:10.3390/ijerph191610206)
Supplement: Supplementary file 1 [file ijerph-19-10206-s001.zip › ijerph-1823266-supplementary/Table_S3_Vector-borne diseases.pdf]

Table S3. Vector-borne diseases related to hydrometeorological hazards in Europe.

**Country**	**Area**	**Outbreaks / epidemics**	**Year**	**Causative pathogens**	**Infectious disease(s) (number of cases)**	**Risk factors**	**Refs.**
RO	Southeastern part	1	Summer of 1996	West Nile virus (WNV)	393 cases of laboratory-confirmed West Nile meningoencephalitis	Specific residence characteristics (presence of mosquitoes indoors and in flooded basements of apartment buildings), sewage-contaminated water from poorly maintained plumbing	[106]
CZ	Moravia	1	July 1997	WNV	13 individuals: neutralizing antibodies against WNV (West Nile fever: 2 confirmed and 3 probable cases)	Abrupt increase of mosquito population density in the flood-affected area	[107]
CZ	Bohemia Prague Vltava Labe Rivers	1	August 2002	Tahyna virus (TAHV)	"Valtice fever"	High mosquito numbers and floodplain forests	[109]
FR	Montpellier	1	2014	Chikungunya virus (CHIKV)	Chikungunya virus infection	Extreme rainfall in late September and early October	[111]

						Increase of mosquito population growth and abundance	
IT	Northern part		2010-2015	WNV	WNV infection	High temperatures (5-6 weeks before WNV diagnosis) Increased precipitation (1–4 weeks before WNV diagnosis)	[112]
GR	Central Macedonia (Northern Greece)	1	2010	WNV	WNV infection	Increased precipitation high temperatures	[110]
